# The MITF regulatory network in melanoma

**DOI:** 10.1111/pcmr.13053

**Published:** 2022-07-09

**Authors:** Jagat S. Chauhan, Michael Hölzel, Jean‐Philippe Lambert, Francesca M. Buffa, Colin R. Goding

**Affiliations:** ^1^ Ludwig Institute for Cancer Research, Nuffield Department of Clinical Medicine University of Oxford Headington Oxford UK; ^2^ Institute of Experimental Oncology University Hospital Bonn, University of Bonn Bonn Germany; ^3^ Department of Molecular Medicine and Cancer Research Centre Université Laval Quebec Quebec Canada; ^4^ CHU de Québec Research Center, CHUL Quebec Quebec Canada; ^5^ Endocrinology – Nephrology Axis CHU de Québec – Université Laval Research Center Quebec City Quebec Canada; ^6^ Department of Oncology University of Oxford Headington Oxford UK

**Keywords:** beta‐catenin, melanoma, MITF, notch, tumor immune infiltration

## Abstract

Bidirectional interactions between plastic tumor cells and the microenvironment critically impact tumor evolution and metastatic dissemination by enabling cancer cells to adapt to microenvironmental stresses by switching phenotype. In melanoma, a key determinant of phenotypic identity is the microphthalmia‐associated transcription factor MITF that promotes proliferation, suppresses senescence, and anticorrelates with immune infiltration and therapy resistance. What determines whether MITF can activate or repress genes associated with specific phenotypes, or how signaling regulating MITF might impact immune infiltration is poorly understood. Here, we find that MITF binding to genes associated with high MITF is via classical E/M‐box motifs, but genes downregulated when MITF is high contain FOS/JUN/AP1/ATF3 sites. Significantly, the repertoire of MITF‐interacting factors identified here includes JUN and ATF3 as well as many previously unidentified interactors. As high AP1 activity is a hallmark of MITF^Low^, invasive, slow‐cycling, therapy resistant cells, the ability of MITF to repress AP1‐regulated genes provides an insight into how MITF establishes and maintains a pro‐proliferative phenotype. Moreover, although β‐catenin has been linked to immune exclusion, many Hallmark β‐catenin signaling genes are associated with immune infiltration. Instead, low MITF together with Notch signaling is linked to immune infiltration in both mouse and human melanoma tumors.

**Significance**

Microenvironment‐driven phenotypic heterogeneity in melanoma influences the outcome of conventional and immunotherapies. The results highlight how MITF, which is associated with a more proliferative/differentiated phenotype, may suppress the activity of genes regulated by AP1, a driver of the undifferentiated/invasive phenotype. They also reveal how the MITF network can be used to predict survival, and how Notch signaling is associated with immune infiltration. Overall we provide a fundamental insight into how phenotypic heterogeneity in melanoma is established and maintained, and into the microenvironment associated with immune infiltration.

## 1 INTRODUCTION

Over recent years, it has been increasingly recognized that cancer progression and therapeutic resistance are driven in large part by the bidirectional interplay between cancer cell plasticity and the tumor microenvironment (Chaffer et al., 2016; Flavahan et al., 2017; Rambow et al., 2019). In response to signals from the microenvironment, including hypoxia, nutrient limitation, inflammatory signaling and therapies, cancer cells adopt specific phenotypes compatible with their survival (García‐Jimenéz & Goding, 2019); despite all cells within a tumor containing the same driver mutations, each tumor may exhibit considerable phenotypic heterogeneity, with some cells actively proliferating while others may be cell cycle arrested or slow cycling and exhibit an invasive or dormant phenotype. Importantly, some phenotypic states are resilient to therapies including targeted or immunotherapies (Marine et al., 2020). Understanding the molecular mechanisms underpinning the generation of phenotypic diversity in response to a changing microenvironment, the relationship of phenotype to therapeutic response, and the characterization of the range of phenotypes present before during and after treatment is critically important if effective anticancer therapies are to be developed.

Melanoma, one of the most aggressive types of cancer that develops from the pigment‐containing cells known as melanocytes, represents an excellent model to understand the molecular events that drive intra‐tumor phenotypic heterogeneity. Since the genetic basis for melanoma initiation has been largely defined (Bennett, 2015; Shain & Bastian, 2016), attention has been increasingly focused on determining the extent and consequences of phenotypic heterogeneity. Most notably, multiple phenotypic states have been identified (Rambow et al., 2019), including those associated with therapy resistance, for example those resistant to immunotherapies or during minimal residual disease following targeting of activated BRAF (Rambow et al., 2018), the primary driver of around 50% of melanomas (Davies et al., 2002). Remarkably, different phenotypic states within human tumors, or those observed in mouse models of the disease, can be reflected within the range of melanoma cell lines available (Tsoi et al., 2018; Wouters et al., 2020), with the caveat that, by definition, cell lines must proliferate, whereas some phenotypic states in vivo may be quiescent. Importantly, different melanoma phenotypes are not fixed, but can undergo transitions in vivo (Hoek, Eichhoff, et al., 2008), a process termed phenotype‐switching (Hoek & Goding, 2010).

Key to understanding phenotype‐switching in melanoma is the microphthalmia‐associated transcription factor MITF (Goding & Arnheiter, 2019), first identified because of its critical role in promoting survival of migrating melanoblasts in development (Hodgkinson et al., 1993). MITF can promote survival, differentiation, and proliferation, and suppress melanoma invasion and senescence (Goding & Arnheiter, 2019). Its central role in melanocyte biology is underpinned by its ability to control a wide variety of cellular processes (Goding & Arnheiter, 2019) including expression of the *ABCB5* transporter (Louphrasitthiphol, Chauhan, & Goding, 2020) and pigmentation‐associated genes (a differentiation function) and genes implicated in cell‐cycle progression (Carreira et al., 2005; Carreira et al., 2006; Du et al., 2004; Loercher et al., 2005; McGill et al., 2006) and metabolism including the TCA cycle (Louphrasitthiphol et al., 2019), mitochondrial biogenesis (Haq et al., 2013; Vazquez et al., 2013) and fatty acid saturation (Vivas‐Garcia et al., 2020). Importantly, MITF has emerged as a key driver and marker of melanoma phenotype, and its mRNA expression and translation is suppressed by a wide range of microenvironmental cues including hypoxia (Cheli et al., 2012; Louphrasitthiphol et al., 2019; Widmer et al., 2013), nutrient limitation (Falletta et al., 2017; Ferguson et al., 2017) and inflammatory signaling (Falletta et al., 2017; Riesenberg et al., 2015), many of which converge on translation reprogramming via phosphorylation of eIF2α (Falletta et al., 2017). For example, while MITF^High^ cells are proliferative (McGill et al., 2006; Widlund et al., 2002) or differentiated (Carreira et al., 2005; Loercher et al., 2005), MITF^Low^ cells tend to be more invasive (Carreira et al., 2006), exhibit enhanced resistance to BRAF inhibition (Konieczkowski et al., 2014; Muller et al., 2014), and have a higher tumor‐forming capacity (Cheli et al., 2011).

Notwithstanding previous attempts to define the repertoire of genes regulated by MITF (Hoek, Schlegel, et al., 2008; Louphrasitthiphol, Siddaway, et al., 2020; Strub et al., 2011; Verfaillie et al., 2015; Webster et al., 2014), the existing signatures and known targets are not sufficient to explain the broad and key role of MITF in melanocyte development, melanoma progression, and drug resistance. Notably, a relatively small number of well‐characterized MITF target genes have been reported (Cheli et al., 2010; Goding & Arnheiter, 2019). Indeed, MITF chromatin immunoprecipitation followed by high throughput sequencing (ChIP‐seq) analysis combined with gene expression data from two independent studies (Strub et al., 2011; Verfaillie et al., 2015) identified only 54 target genes in common, a number reduced to just 9 when including the genes identified by Hoek, Schlegel, et al. (2008). It is also unclear from analysis of MITF target genes why low MITF expression is related to an increase in tumor‐infiltrating immune cells nor how in the same cells it may repress or activate different sets of genes. To address these questions, we set out to undertake a comprehensive analysis of MITF targets and associated phenotypes, using genomics and transcriptomic data from cell lines and clinical samples, chromatin immunoprecipitation‐sequencing (ChIP‐seq) data and mass spectrometry.

## 2 MATERIALS AND METHODS

Gene expression (RNA‐seq) data from the TCGA Skin Cutaneous Melanoma (SKCM) cohort was downloaded from the cBioportal for Cancer Genomics (http://www.cbioportal.org) using CGDS‐R [https://cran.r‐project.org/web/packages/cgdsr/index.html] following TCGA guidelines (http://cancergenome.nih.gov/publications/publicationguidelines). We used preprocessed normalized RSEM (RNA‐Sequencing by Expectation Maximization) data. The R packages limma and EdgeR were used for identification of differentially expressed genes (DEGs) between low and high MITF phenotypes. We used overlapping list of genes from Limma and EdgeR for final set of DEGs. Genes with false discovery rate <0.05 and *p*‐value <.05 were considered differentially expressed. RSEM values <1 were set to 1 to avoid negative expression values upon log2 transformation if necessary. The moving average expression of individual genes and gene sets was calculated with a sample window size of *n* = 20. Gene expression profiles of gene and gene sets are displayed as heatmap using Pheatmap (https://cran.r‐project.org/web/packages/pheatmap/index.html). For ChIP‐seq data analysis, we used ChIP‐seq peaks from our previous study. The ChIP‐seq peaks were annotated using HOMER (Heinz et al., 2010) that was also used to detection and examine motif enrichment among peak regions. We used ChIPseeker package for ChIP‐seq peak annotation and visualization (Yu et al., 2015).

### 2.1 Co‐expression analysis and gene ontology

We calculated Spearman's based auto‐correlation for predicted up and down targets. We also used weighted gene correlation network analysis (WGCNA) (Langfelder & Horvath, 2008) to identify subtarget class. Gene ontology enrichment of differentially expressed MITF targets were performed using the R package clusterProfiler and ToppFunn module of the ToppGene suite (Chen et al., 2009) with a Benjamini‐Hochberg multiple testing adjustment and an FDR cut‐off of 0.05, using all expressed genes as a background control. The results were visualized as dot plots using the clusterProfiler and ggplot2 R packages.

### 2.2 Immune cell fraction

All SKCM TCGA samples were processed by xCell (Aran et al., 2017) to estimate relative enrichment of 64 stroma and immune cell types, and we then compared between low MITF (100 samples) and high MITF (100 samples) by Wilcoxon test to find the most significant cell types.

### 2.3 Gene set analysis for TCGA data

Gene Set Variation analysis (GSVA) (Hänzelman et al., 2013) was used to measure the sample‐wise enrichment score for each gene modules and immune gene signatures. The generated GSVA scores of each gene set were compared between low and high MITF samples and displayed in the heatmaps. The used default parameters in GSVA package.

### 2.4 Survival analysis

Cox regression‐based survival analysis is applied to analyze clinical factors and each differential expression gene. Each up‐ and down‐ target with an FDR <0.05 were selected as input for LASSO regression using the glmnet package (https://cran.r‐project.org/web/packages/glmnet/index.html) in R 3.3.1. “Cox” was set as the family in the model. Ten‐fold cross‐validation was performed using the cv.glmnet function to select lambda minimum to give the minimum cross‐validated error. The resulting 19 genes with coefficients were used to calculate a predictive index for each patient.

### 2.5 Analysis of mouse melanomas

Generation of the two Illumina Murine Beadchips WG6 v. 2.0 based gene expression datasets (GSE40213, GSE99925) from ACT untreated and treated HCmel3 mouse melanomas were described previously (Landsberg et al., 2012; Reinhardt et al., 2017). For re‐analysis, raw microarray data extracted from the Illumina BeadStudio software were imported into the R statistical programming environment and the Bioconductor platform using the beadarray package. Variance stabilization (vsn2) and normalization were performed followed by quality assessment of the fit. Expression data were log2 transformed. In essence, the combined dataset reflects a spectrum of syngeneic HCmel3 mouse melanomas with different degrees of immune cell infiltration as a surrogate to the situation found in human melanomas from TCGA database. Notch and Wnt signature genes determined by the analysis of human melanomas as well as *Mitf* were correlated (Pearson correlation coefficients) with expression of the averaged mouse immunome signature and visualized by heatmap. For the mouse immunome signature, homologous genes of the human immunome signature genes were identified and selected.

### 2.6 Generation of MITF tagged cell lines and culture conditions

Flp‐In T‐REx HEK293 cells (female; Invitrogen) were grown in DMEM +5% FBS and 5% calf serum containing penicillin and streptomycin. Cells were grown at 37°C in 5% CO_2_. BirA*‐FLAG‐MITF was stably expressed in Flp‐In T‐REx HEK293 as previously described (Lambert et al., 2014). Flp‐In T‐REx HEK293 cells expressing BirA*‐FLAG fused to a nuclear localization sequence (NLS) were used as negative controls for the BioID experiments and processed in parallel to the bait proteins. Two 150‐mm plates of stable cell lines were selectively grown in the presence of 200 μg/ml hygromycin up to 80% confluence before the expression was induced via 1 mg/ml tetracycline for 24 h. Then, 50 mM biotin was added for 4 h before harvesting the cells. To do so, cells were pelleted at low speed, washed with ice‐cold phosphate‐buffered saline (PBS), and frozen at −80°C until BioID purification.

### 2.7 Proximity‐dependent biotinylation mass spectrometry

Cell pellets from two 150‐mm plates were pelleted, frozen, and thawed in 1.5 ml ice cold RIPA buffer containing 50 mM Tris–HCl (pH 7.5), 150 mM NaCl, 1% NP‐40, 1 mM EDTA, 1 mM EGTA, 0.1% SDS and 0.5% sodium deoxcycholate. PMSF (1 mM), DTT (1 mM) and Sigma‐Aldrich protease inhibitor cocktail (P8340, 1:500) were added immediately before use. The lysates were sonicated using a QSONICA 125 W sonicator equipped with 1/8″ probe. Benzonase (100 units) was added and the lysates were incubated at 4°C for 1 h with rotation. The lysates were centrifuged at 20,817 *g* for 20 min at 4°C. For each sample, 60 ml of streptavidin‐Sepharose bead slurry (Cytiva, Cat 17‐5113‐01) was prewashed three times with 1 ml of lysis buffer by pelleting the beads with gentle centrifugation and aspirating off the supernatant before adding the next wash. Biotinylated proteins were captured on prewashed streptavidin beads for 3 h at 4°C with rotation. The beads were gently pelleted and then washed twice with 1 ml RIPA buffer and three times with 1 ml 50 mM ammonium bicarbonate (pH 8.0). Following the final wash, the beads were pelleted and any excess liquid was aspirated off. Beads were resuspended in 100 μl of 50 mM ammonium bicarbonate, and 1 μg of trypsin solution was added. The samples were incubated overnight at 37°C with rotation and then an additional 1 μg of trypsin was added, followed by further incubation for 2–4 h. The beads were pelleted and the supernatant was transferred to a fresh tube. The beads were rinsed twice with 100 μl HPLC‐grade water, and the wash fraction was combined with the supernatant. The peptide solution was acidified with 50% formic acid to a final concentration of 2% and the samples were placed in a Speedvac to dry. Tryptic peptides were resuspended in 25 ml 5% formic acid and stored at −80°C until mass spectrometry analysis.

### 2.8 Mass spectrometry acquisition using TripleTOF 5600 mass spectrometers

Each sample (5 μl) was directly loaded at 400 nl/min onto an equilibrated HPLC column. The peptides were eluted from the column over a 90‐min gradient generated by a NanoLC‐Ultra 1D plus (Eksigent, Dublin CA) nano‐pump and analyzed on a TripleTOF 5600 instrument (AB SCIEX, Concord, Ontario, Canada). The gradient was delivered at 200 nl/min starting from 2% acetonitrile with 0.1% formic acid to 35% acetonitrile with 0.1% formic acid over 90 min followed by a 15‐min clean‐up at 80% acetonitrile with 0.1% formic acid, and a 15‐min equilibration period back to 2% acetonitrile with 0.1% formic acid, for a total of 120 min. To minimize carryover between each sample, the analytical column was washed for 3 h by running an alternating sawtooth gradient from 35% acetonitrile with 0.1% formic acid to 80% acetonitrile with 0.1% formic acid, holding each gradient concentration for 5 min. Analytical column and instrument performance were verified after each sample by loading 30 fmol of bovine serum albumin (BSA) tryptic peptide standard (Michrom Bioresources Fremont, CA) with 60 fmol a‐casein tryptic digest and running a short 30 min gradient. TOF MS calibration was performed on BSA reference ions before running the next sample to adjust for mass drift and verify peak intensity. The instrument method was set to data‐dependent acquisition (DDA) mode, which consisted of one 250 ms MS1 TOF survey scan from 400 to 1300 Da followed by 20,100 ms MS2 candidate ion scans from 100 to 2000 Da in high sensitivity mode. Only ions with a charge of 2+ to 4+ that exceeded a threshold of 200 cps were selected for MS2, and former precursors were excluded for 10 s after one occurrence.

### 2.9 Data‐dependent acquisition MS analysis

Mass spectrometry data were stored, searched, and analyzed using the ProHits laboratory information management system (LIMS) platform (Liu et al., 2016). Within ProHits, AB SCIEX WIFF files were first converted to an MGF format using WIFF2MGF converter and to an mzML format using ProteoWizard (v3.0.4468) and the AB SCIEX MS Data Converter (v.1.3 beta). The mzML files were then searched using Mascot (version 2.3.02) and Comet (version 2012.02 rev.0). The spectra were searched with the RefSeq database (version 57, January 30th, 2013) acquired from NCBI against a total of 72,482 human and adenovirus sequences supplemented with common contaminants from the Max Planck Institute (http://lotus1.gwdg.de/mpg/mmbc/maxquant_input.nsf/7994124a4298328fc125748d0048fee2/$FILE/contaminants.fasta) and the Global Proteome Machine (GPM; https://www.thegpm.org/crap/index.html). For TripleTOF 5600 files, the database parameters were set to search for tryptic cleavages, allowing up to two missed cleavage sites per peptide with a mass tolerance of 40 ppm for precursors with charges of +2 to +4 and a tolerance of 0.15 amu for fragment ions. Deamidated asparagine and glutamine and oxidized methionine were allowed as variable modifications. The results from each search engine were analyzed through the Trans‐Proteomic Pipeline (version 4.6 OCCUPY rev 3) via the iProphet pipeline (Shteynberg et al., 2011). SAINTexpress version 3.6.1 (Teo et al., 2014) was used as a statistical tool to calculate the probability value of each potential protein–protein interaction compared to background contaminants using default parameters. Two unique peptide ions and a minimum iProphet probability of 0.95 were required for protein identification prior to SAINTexpress.

### 2.10 Experimental design and statistical rationale for MS experiments

For each sample type, two biological replicates were processed independently. These were analyzed with negative controls in each batch of samples processed. The order of samples as they were acquired on the LC–MS/MS system was randomized. Statistical scoring was performed against three negative controls, composed of NLS‐BirA*‐FLAG Flp‐In T‐REx HEK293 controls using Significance Analysis of INTeractome (Teo et al., 2014; SAINTexpress version 3.6.1). The average SAINTexpress score was used to determine the Bayesian FDR, which requires a high confidence interaction to be detected across biological replicates, to pass our 1% FDR significance threshold.

## 3 RESULTS

**FIGURE 1:**
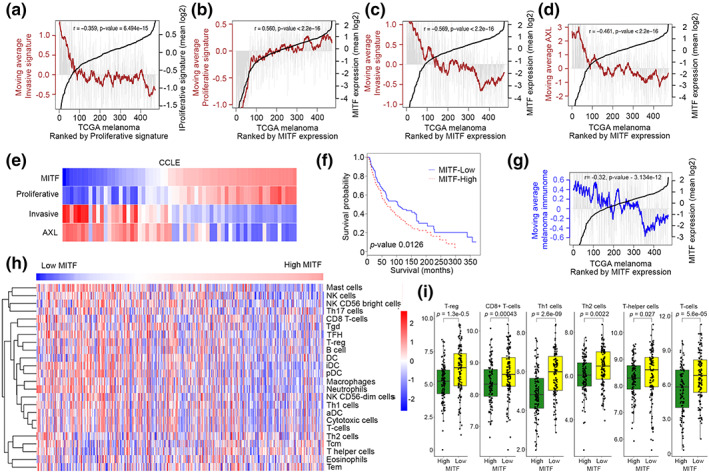
MITF expression anticorrelates with invasion and immune infiltration. (a) the 473 melanomas in the TCGA cohort were ranked by expression of the Verfaillie et al proliferative signature (black line). Gray bars indicate average expression of Verfaillie et al.’s invasive signature in each of the tumors, and the brown line represents the moving average of the invasive signature over each 20 tumors. (b–d) TCGA melanomas ranked by MITF expression and the moving average of the Verfaillie proliferative (b) or invasive (c) signatures, or AXL (d) in each represented by the gray bars and moving average in brown. (e) Heatmap showing the CCLE melanoma cell lines ranked by MITF expression showing relative expression in each cell line of AXL or the Verfaillie et al.’s invasive or proliferative gene expression signatures. (f) Kaplan–Meier plot showing relative survival for MITF^High^ versus MITF^Low^ TCGA melanomas; 230 samples for each. (g) TCGA melanomas ranked by MITF expression with a gene expression signature corresponding to the melanoma “immunome” shown in each tumor as gray bars and the moving average in blue. (h) TCGA melanomas ranked by MITF expression showing relative expression of gene expression signatures corresponding to the indicated immune cell types (dendritic cells [DCs], immature DCs [iDCs], activated DCs [aDCs], eosinophils, mast cells, macrophages, natural killer cells [NKs], NK CD56^dim^ cells, NK CD56^bright^ cells, and neutrophils) and adaptive immune cells (B, T helper 1 [Th1], Th2, T gamma delta [Tγδ], CD8^+^ T, T central memory [Tcm], T effector memory [Tem], and T follicular helper [Tfh] cells). (i) box and whisker plots showing relative expression of different immune expression signatures in the MITF^High^ versus MITF^Low^ melanomas (top/bottom 25%) in the TCGA melanoma cohort

Depletion of MITF induces invasion and a G1 cell cycle arrest (Carreira et al., 2006), indicating that MITF promotes proliferation and suppresses metastatic dissemination. If so, MITF mRNA expression should broadly relate to previously determined melanoma‐associated proliferative or invasive gene expression signatures (Verfaillie et al., 2015) that are inversely correlated in the 473 tumor samples in the TCGA melanoma cohort (Figure 1a). As a first step, we initially examined the RNA‐seq data in the TCGA melanoma cohort (*n* = 473) for correlation between MITF mRNA expression and that of the Verfaillie et al. (2015) proliferative and invasive gene expression signatures that we chose so as to enable comparisons to be made with our previous publications (Falletta et al., 2017; Louphrasitthiphol et al., 2019; Vivas‐Garcia et al., 2020). As expected, ranking tumors by MITF expression revealed a significant correlation with the proliferative gene expression signature that was especially marked when MITF is low (Figure 1b), and an anticorrelation with an invasive gene expression signature (Figure 1c). Similar results were also obtained using Hoek et al.’s proliferative and invasive signatures (Figure S1). Since the TCGA melanomas frequently include a significant proportion of nonmelanoma cells such as fibroblasts or infiltrating immune cells (https://www.cancer.gov/tcga), we also verified the correlations between MITF expression and the invasive and proliferative gene expression signatures using the gene array data from the Cancer Cell Line Encyclopedia panel of melanoma cells. Using AXL, that significantly anticorrelates with MITF in the TCGA (Figure 1d), as an independent marker, the results (Figure 1e) confirmed that MITF correlates with the proliferative gene expression signature and anticorrelates with the invasive signature. Notably, the MITF^Low^ tumors exhibited a moderate survival advantage compared to the MITF^High^ melanomas (Figure 1f). Using a set of 380 genes corresponding to 24 immune cell types, originally developed for application to colorectal cancer (Bindea et al., 2013) but later applied to melanoma (Nsengimana et al., 2018), we were also able to show that melanomas with low MITF expression were enriched in the melanoma “immunome,” while MITF^High^ tumors exhibited reduced immune infiltration (Figure 1g). The relationship between MITF and tumor infiltration by the 24 immune cell types that together comprise the immunome, including mast cells, macrophages, NK cells, neutrophils, and T‐cells, including T‐helper (CD4^+^) cells that play an important role in activation of cytotoxic CD8+ T‐cells is shown in Figure 1h and i. Although MITF clearly anticorrelates with immune infiltration into melanomas, whether MITF plays a role in vivo in regulating immune cell infiltration is not understood. Nevertheless, these data confirm work from several laboratories suggesting that MITF is a key regulator that dictates melanoma heterogeneity.

### 3.1 Gene expression as a proxy for MITF activity

**FIGURE 2:**
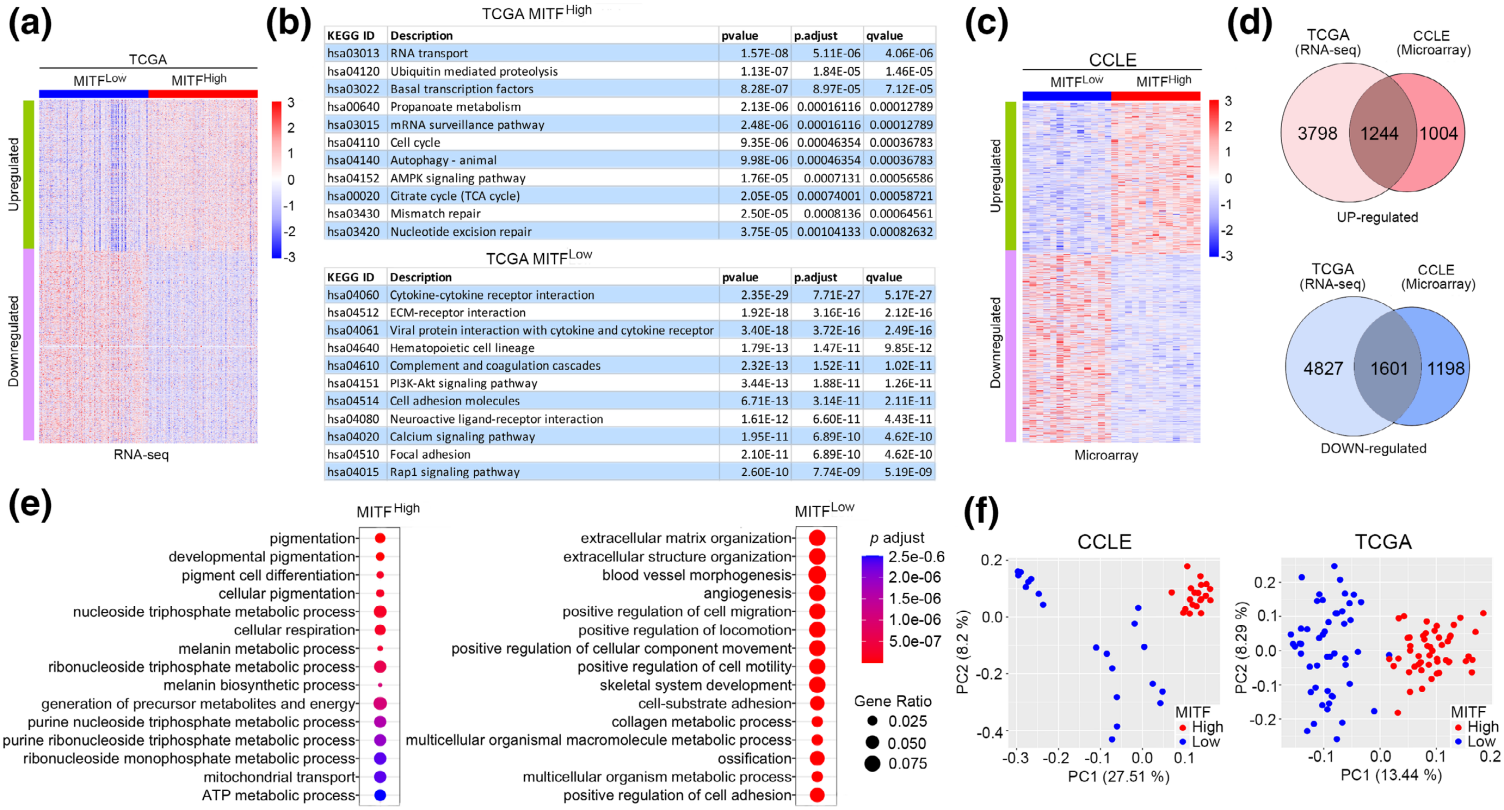
MITF correlated genes. (a) Heatmap showing differential expression in MITF^High^ versus MITF^Low^ TCGA melanomas. (b) KEGG pathways associated with genes differentially expressed between MITF^High^ and MITF^Low^ TCGA melanomas. (c) Heatmap showing differential expression in 13 MITF^High^ versus 13 MITF^Low^ CCLE melanoma cell lines. (d) Venn diagrams showing the overlap between the differentially expressed genes in the MITF^High^ versus MITF^Low^ classes of the TCGA and CCLE. (e) Biological processes associated with the common differentially expressed genes associated with the MITF^High^ versus MITF^Low^ classes. (f) Principal component analysis of the MITF^High^ versus MITF^Low^ classes TCGA and CCLE datasets

To derive a signature of MITF activation in cell lines and clinical samples, we needed to understand if the expression of MITF is a reasonable proxy for activation of MITF as this is not necessarily the case for transcription factors (Brent, 2016). To this end, we used RNA‐seq data from the 473 sample TCGA melanoma cohort to identify differentially expressed genes (DEGs) with an adjusted *p*‐value (*q*‐value) ≤ 0.05 whose expression was significantly different between the top and bottom 25% when ranked by MITF expression. This cut‐off was chosen since the top and bottom 25% of melanomas ranked by MITF include the highest and lowest melanomas exhibiting an invasive gene expression signature and exclude those where the invasive signature is not changing substantially (see Figure 1c). We detected 5042 genes significantly upregulated in the MITF^High^ melanomas compared to those ranked as MITF^Low^, and 6428 genes down‐regulated (*p* = <.05; Figure 2a). The biological processes associated with high or low MITF activity within the TCGA melanoma cohort were revealed using the Kyoto Encyclopedia of Genes and Genomes (KEGG) pathway analysis (Figure 2b). This analysis indicated that MITF^High^ melanomas exhibited an elevated number of processes known to be related to MITF function including the cell cycle, autophagy, the TCA cycle, and DNA damage repair, as well as mRNA transport and surveillance, AMPK signaling, and metabolism of the short chain fatty acid propionate. By contrast, the genes associated with MITF^Low^ melanomas were associated with inflammatory signaling, PI3K/AKT signaling, cell adhesion, and FAK signaling. These observations are consistent with a previous study that reported that MITF can reprogram the extracellular matrix and focal adhesions (Dilshat et al., 2021).

While these analyses give a good indication of the processes correlated to MITF activity in vivo, any correlation with MITF expression should be independent of dataset or technique used to generate the data. Moreover, tumor samples within the TCGA melanoma cohort will also contain nonmelanoma cells that may be present in varying ratios in the MITF^High^ versus MITF^Low^ tumor samples. Gene expression in these noncancer cell types may therefore confound the analysis of differential gene expression associated with melanoma cells. To control for this, we examined the publicly available melanoma/skin Cancer Cell Line Encyclopedia gene expression data to obtain 2248 upregulated and 2799 downregulate genes in 13 high MITF vs 13 low MITF‐expressing cell lines (Figure 2c). Of the up‐regulated DEGs, 1244 were common between the TCGA and CCLE datasets (Figure 2d) with 1601 common DEGs in the downregulated set. Notably, the common upregulated geneset included many known MITF targets implicated in melanocyte differentiation (e.g., DCT, MLANA, PMEL, TYR, TYRP1, ABCB5) and cell proliferation (e.g., CDK2, PPARGC1A) (Table S1). Gene Ontology (GO) analysis of the genes common to the TCGA and CCLE in the MITF‐high and ‐low classes (Figure 2e) revealed as expected that MITF is highly positively correlated with expression of genes implicated not only in pigmentation and differentiation, but also in anabolic processes such as nucleoside triphosphate production, respiration, and generation of precursor metabolites, consistent with MITF promoting proliferation. By contrast, low levels of MITF were related to cell migration and production of extracellular matrix such as collagen, consistent with MITF repressing ECM expression (Dilshat et al., 2021). In addition, angiogenesis was downregulated in the MITF^High^ melanomas. This is consistent with blood vessel formation being driven by a need to supply nutrients and oxygen, and low nutrient supply or hypoxia being linked to low MITF and invasion as described previously (Cheli et al., 2012; Falletta et al., 2017; Feige et al., 2011; Ferguson et al., 2017; Louphrasitthiphol et al., 2019; Widmer et al., 2013). Principal component (PC) analysis (Figure 2f) confirmed using both the TCGA and CCLE datasets that MITF is a main explanatory variable for variation of gene expression between the MITF^High^ and MITF^Low^ tumors or cell lines.

**FIGURE 3:**
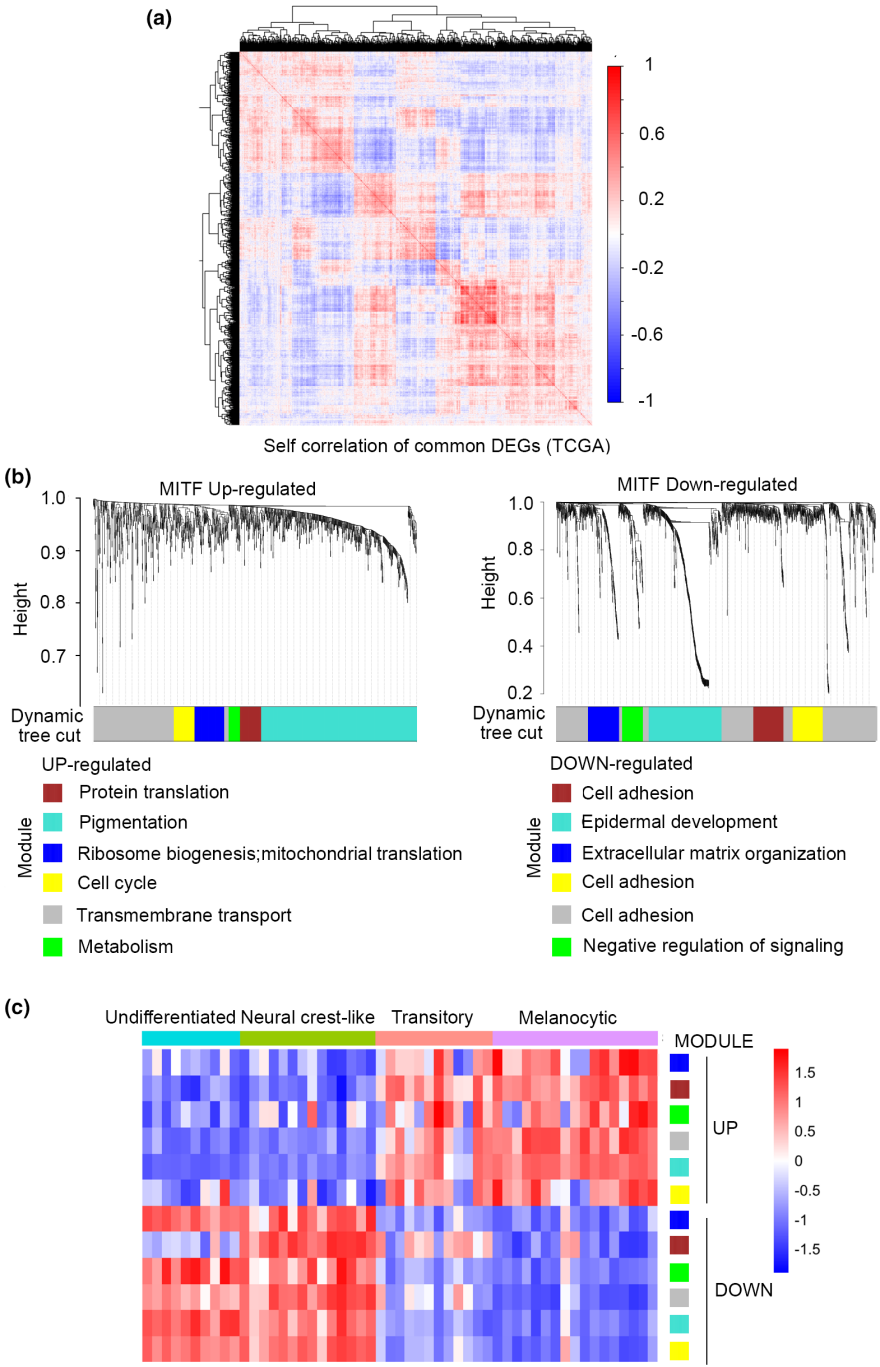
Distinct gene expression modules are associated with MITF^High^ and MITF^Low^ gene expression. (a) Self‐correlation analysis of the TCGA melanoma gene expression data of common differentially expressed genes (DEGs) between the TCGA and CCLE datasets. (b) WGCNA identifies distinct modules within the differentially expressed genes associated with high or low MITF in common between the TCGA and CCLE datasets. (c) Expression of each module in the Tsoi et al data set of 53 melanoma cell lines clustered into distinct phenotypic classes

Although the identification of DEGs provides some insight into the biological processes associated with high or low MITF expression, it was likely that the repertoire of genes identified would contain subsets whose expression would be tightly coordinated by signals associated with specific conditions encountered within the tumor microenvironment. To investigate this possibility, we first used the DEGs for self‐correlation analysis within the TCGA dataset. The results (Figure 3a) indicated that the genes fell into a number of distinct clusters suggesting there is a degree of organization of gene expression of genes associated with MITF expression in addition to MITF activity. This preliminary observation then prompted us to apply Weighted Gene Correlation Network Analysis (WGCNA), a systems biology tool used to identify sets of genes whose expression is tightly correlated and therefore are likely to be co‐regulated by the same signals/transcription factors. WGCNA identified six gene modules of coexpressed genes for the upregulated genes (Figure 2b) and the same number of modules for the downregulated genes. A list of genes in each of the modules identified for the up‐ or downregulated gene sets are provided in Table S2. GO analysis of the genes within each module provided additional information on the biological processes associated with MITF function. For the modules identified for the upregulated gene set, GO analysis (Figure S2) revealed the Turquoise module contained genes linked to pigmentation, including (Table S2) the well‐characterized differentiation‐associated MITF targets DCT, TYR, MLANA, PMEL, and ABCB5 (Louphrasitthiphol, Chauhan, & Goding, 2020) as well as other known MITF targets such as SCD (Vivas‐Garcia et al., 2020) and CDK2 (Du et al., 2004) implicated in controlling fatty acid saturation and the cell cycle respectively, as well as a range of solute carriers and genes implicated in lysosome biogenesis. Additional modules were linked to protein translation (Brown), ribosome biogenesis (Blue), and the cell cycle (Yellow). In addition, the Grey module was associated with membrane transport and the Green with metabolism. Although many of these biological process have already been identified as regulated by MITF, protein translation and ribosome biogenesis have not previously been linked to MITF function.

By contrast, the modules associated with the set of genes downregulated in MITF^High^ melanomas were predominantly linked to cell adhesion and motility. In addition, although not assigned to a specific module, a range of genes linked to inflammatory gene expression such as *IL1B*, *IL8*, *IL4I1,* and *CCL5* were also identified in the downregulated genes (Table S2), consistent with previous observations that depletion of MITF leads to an inflammatory secretome (Ohanna et al., 2011).

The differential expression of each module was then confirmed in an independent dataset derived from a published panel of 53 melanoma cell lines that fall into 4 phenotypically distinct states (Tsoi et al., 2018). The results (Figure 3c) indicated that the modules associated with the MITF^High^ and MITF^Low^ tumors were associated with the MITF^High^ transitory and melanocytic phenotype lines, whereas the downregulated modules were primarily expressed in the undifferentiated or neural crest phenotypes. Of note, unlike the other MITF^Low^ modules, the Brown module was less expressed in the mesenchymal phenotype.

### 3.2 Identification of directly bound MITF targets

Since the CCLE dataset is derived from melanoma cell lines, we regarded the genes identified to be commonly up‐ or downregulated between the TCGA and CCLE as representing a gene set likely to include many directly MITF‐regulated genes. However, given that different melanoma cell lines exhibit a highly variable gene expression response to activation of a specific transcription factor (Louphrasitthiphol et al., 2019), it was unlikely that the common genes include the full repertoire MITF targets. To identify a robust set of directly bound and regulated MITF target genes, we next asked how many of the putative up‐ or downregulated targets identified in common between the TCGA and CCLE datasets were potential direct targets based on published (Louphrasitthiphol, Siddaway, et al., 2020) MITF chromatin immunoprecipitation‐sequencing (ChIP‐seq) data.

**FIGURE 4:**
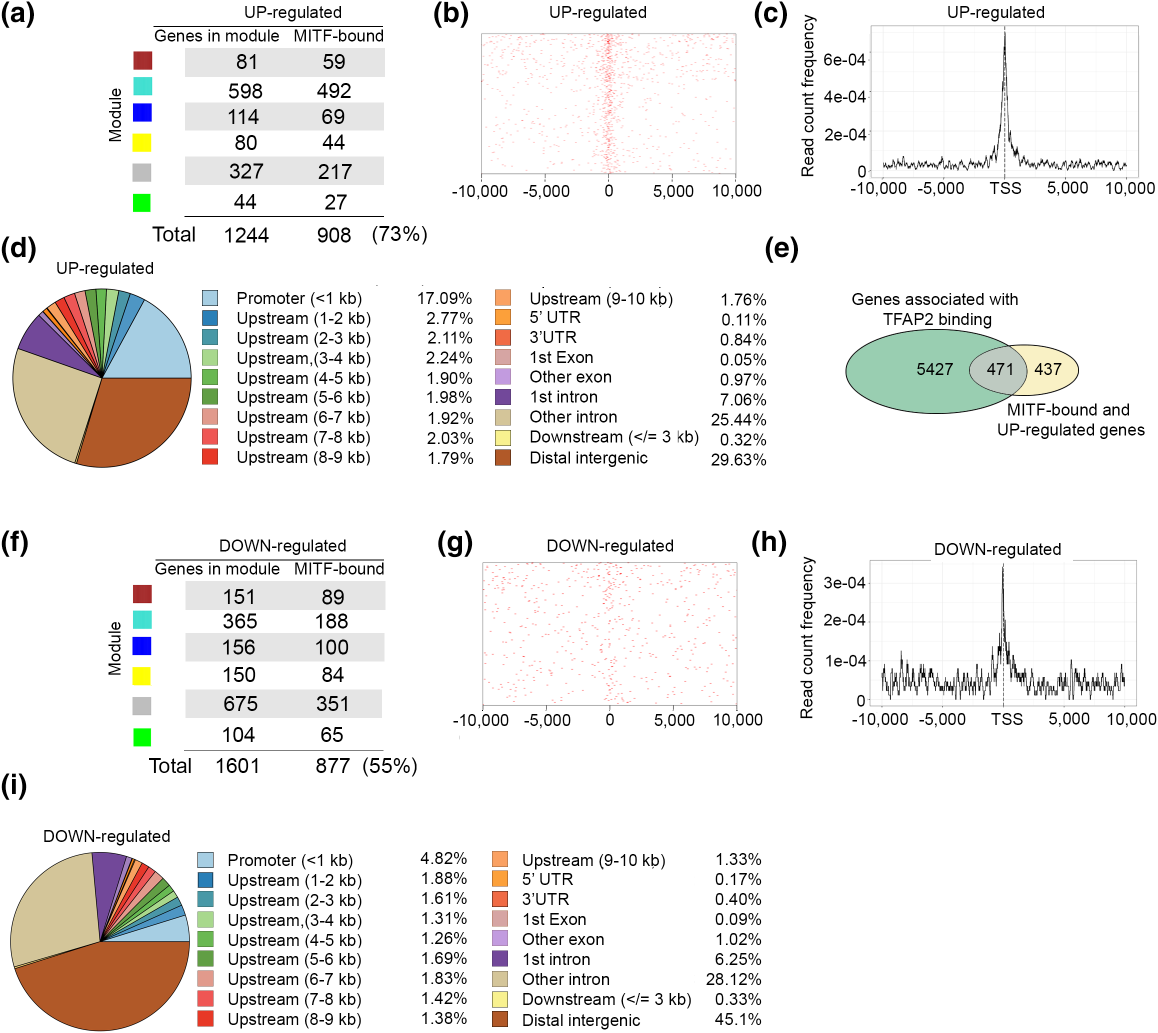
Differential binding between MITF up‐ and down‐regulated genes. (a) Numbers of genes bound by MITF as determined by ChIP‐seq in each module identified by WCGNA in Figure 3b in the positively correlated gene set. (b, c) relative position of the MITF binding site relative to the transcription startsite (TSS) in the positively correlated differentially expressed genes. (d) Genomic distribution of MITF binding sites for MITF bound and positively correlated genes. (e) Venn diagram showing overlap between TFAP2‐bound genes and those bound and positively correlated with MITF expression. (f) Numbers of genes bound by MITF as determined by ChIP‐seq in each module identified by WGCNA in Figure 3b in the negatively correlated gene set. (g, h) relative position of the MITF binding sites relative to the transcription startsite (TSS) in the negatively correlated differentially expressed genes. (i) Genomic distribution of MITF binding sites for MITF bound and negatively correlated genes

To this end, we determined the peak frequency at the genes that positively or negatively correlated with MITF in common between the CCLE and TCGA datasets using the ChIPseeker package with the hg19 genomic annotation. Reads within 5 kb of upstream and downstream of transcription start sites (TSS) were considered. Of the 1244 genes significantly correlating with MITF expression, 908 (73%) contained binding sites for MITF (Figure 4a). Notably, the binding sites were predominantly close to the TSS (Figure 4b,c). The relative genomic distribution of the MITF binding sites is shown in Figure 4d, with over 17% within 1 kb of the TSS, increasing to around 26% within 5 kb. A further 25% were found in introns other than the first intron, and almost 30% in distal intergenic regions. As expected, 9 of the top 10 most significant motifs found in MITF‐correlated genes contained the core CACGTG E‐box, with those with the highest significance also containing the flanking 5′ T or 3′A residue that increases MITF DNA binding affinity (Figure S3A). Note that while the elements identified can be bound by other transcription factors such as USF1 or USF2 that share a bHLH‐LZ DNA binding domain, they would not be able to bind simultaneously with MITF to the same sequence. We also examined sequences within 50 bp of the peak center from the MITF ChIP‐seq to identify any potential cooperating transcription factors. As expected, the results (Figure S3B) again highlighted consensus CAC/TGTG MITF‐binding sites that can also be recognized by other bHLH‐LZ factors such as USF1/2. In addition, we also found recognition motifs for SIX1, ATF3, FOS, and JUN family members, though with greatly reduced *p*‐values compared to the consensus MITF recognition motif. However, the analysis is complicated in that the 8 ‐bp high affinity recognition motif for MITF *TCACGTGA* also contains a partial recognition motif *TGA* for bZIP factors like CREB, ATF3 or FOS and JUN that would become a full consensus if the appropriate 3′ base pairs were present. It is therefore not surprising that at least some MITF recognition sites also contain motifs for bZIP factors. In addition, we do not find statistically significant enrichment for binding motifs for SOX10 (*p* = 1.00E‐01), while co‐occupancy with TFAP2, a transcription factor previously implicated in driving melanocyte differentiation alongside MITF (Seberg et al., 2017), is statistically significant (*p* = 1.00E‐0.2) and TFAP2 binding, identified from published ChIP‐seq data is found in 52% of MITF bound and up‐regulated genes as indicated in Figure 4e.

By contrast, the genes bound by MITF and associated with low MITF expression, that is genes likely to be repressed by MITF, exhibited a fundamentally different pattern. On the 6 modules identified, only 877 (55%) of the total 1601 genes identified were bound by MITF (Figure 4f), and the binding sites exhibited a different pattern relevant to the TSS (Figure 4g, h). Compared to the 17% of binding sites within 1 kb of the TSS for the genes correlated with high MITF, only 4.8% were in this location for the low MITF‐associated genes (Figure 4i). Moreover, while a similar proportion of binding sites was found in introns, the low MITF‐correlated genes exhibited 45% of binding sites in distal intergenic regions, much higher than the 30% for the high MITF‐correlated genes. Most strikingly, although 323 genes contained a CACGTG motif, none of the most significant motifs found associated with the genes anticorrelated with MITF contained an E‐box (Figure S3C). Instead, the most significant binding motifs were associated with TGAC/GTCA sites known to be recognized by ATF3, AP1 (Fos/Jun) or sites recognized by NFκB, NPC, TCF4, OCT4, suggesting that at least for some genes, repression may be indirectly regulated by MITF.

One plausible model to account for the self‐correlation of subsets of potential MITF‐regulated genes is that in addition to MITF, genes in specific modules use distinct transcription factors to drive expression. To investigate this possibility, we examined transcription factor motifs enriched in each module (Figure S4). Among the MITF‐correlated genes, we found enrichment of AP4 binding sites in the green module linked to metabolism, consistent with AP4’s function as a coregulator of MYC targets implicated in metabolism and the cell cycle (Chou et al., 2014; Jackstadt & Hermeking, 2014). We also noted that genes in the yellow module associated with the cell cycle were enriched in binding sites for a number of E2F family members, transcription factors implicated in controlling cell cycle progression. For the gray, blue, and brown modules, no motifs beyond the E‐boxes that would be recognized by MITF stood out, while in the Turquoise module, we noted both E‐boxes and homeo‐domain transcription factor binding sites.

For the downregulated modules, the Yellow and Turquoise were enriched in sites for AP1 and ATF3, related b‐Zip family members, and the latter was also enriched in NFκB sites, while the Brown module contained NFAT, homeo domain, and T‐box family recognition motifs in addition to AP1/ATF3. b‐Zip factor recognition sites as well as T‐elements and homeodomain binding sites were also enriched in other modules. In summary, the repertoire of motifs in genes anticorrelating with MITF were very different from those correlating with MITF, and in some cases different modules tended to have enrichment of different transcription factor motifs.

Collectively, these data suggest that while MITF activates genes via its canonical E‐box recognition motif, it may repress genes either directly or indirectly through long‐range interactions by preventing regulation by a range of transcription factors known to be active in MITF^Low^ melanomas such as AP1 (Riesenberg et al., 2015; Verfaillie et al., 2015) and NFκB (Ohanna et al., 2011). If so, we might expect MITF to interact with transcription factors that drive the gene expression program associated with the dedifferentiated phenotype. To investigate this possibility, we stably expressed MITF tagged with BirA that would biotinylate proteins that interact with MITF. After pull‐down using streptavidin beads, we analyzed the repertoire of interacting factors using mass spectrometry. The results are shown in Table S3 where the interactors above our stringent statistical cutoff include known MITF‐binding proteins such as the MITF‐related factors TFE3 and TFEB, the chromatin remodeling factors CHD7 and SMARCA4, as well as USP11 and TRIM24, all previously identified by Laurette et al. (2015). However, while the majority of MITF‐interacting factors identified in the Laurette et al.’s study are found in our analysis, they fall below the stringent statistical cut‐off. Our analysis also identified the well‐characterized MITF acetyl transferases EP300 and CREBBP as well as a range of novel proteins such as the components of the NCOR complex. We also identify MITF‐interaction with TFAP2 a transcription factor that coregulates melanocyte differentiation‐associated genes alongside MITF and which binds around 50% of MITF up‐regulated genes (Figure 4e). Importantly, JUN, a component of AP1, and ATF3 are also found in the list of significant MITF‐interacting proteins.

### 3.3 MITF target genes and melanoma survival

**FIGURE 5:**
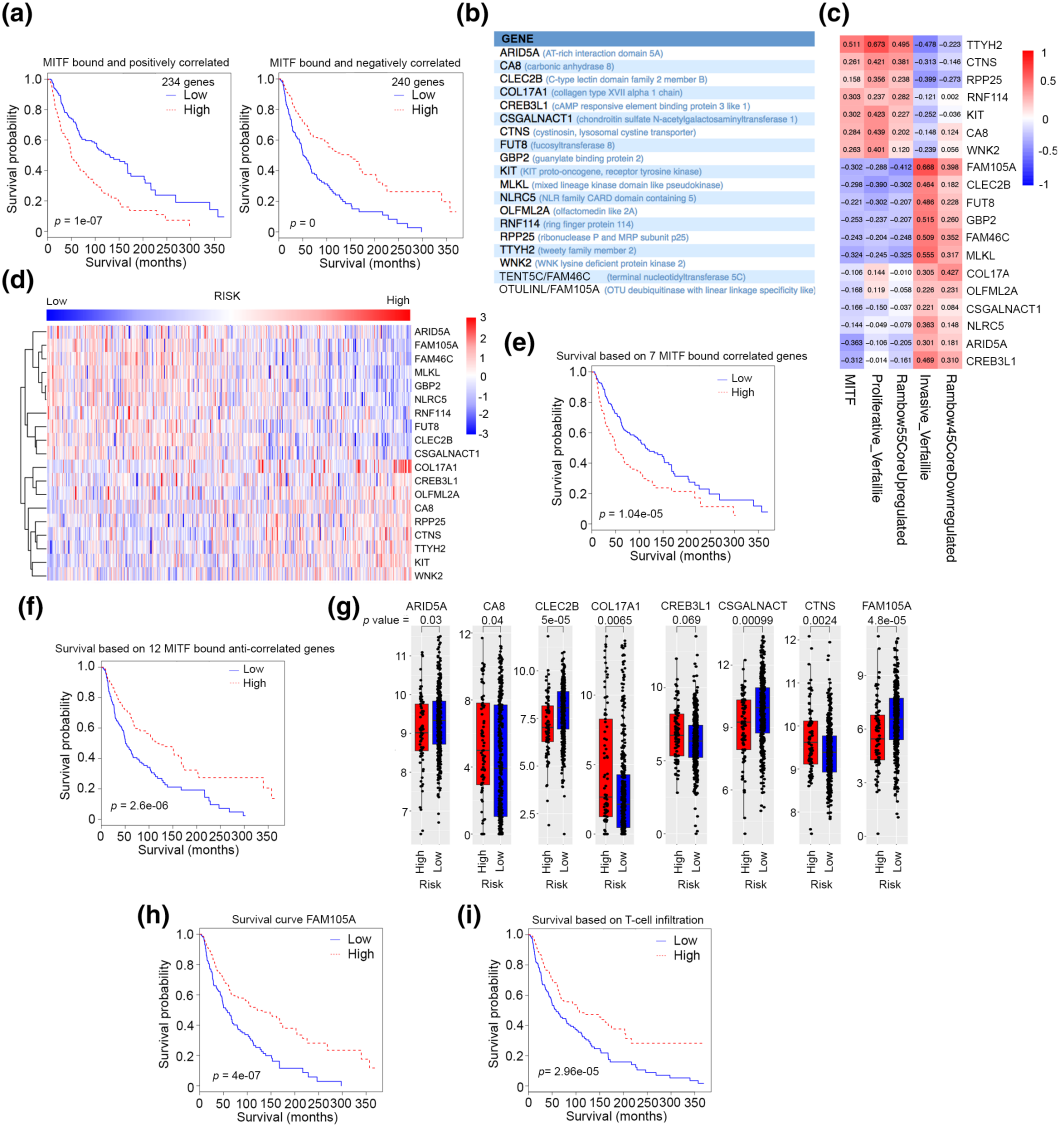
MITF regulated genes and survival. (a) Kaplan–Meier plots showing survival after stratification of the TCGA melanomas by the MITF bound and positive or negatively correlated differentially expressed genes. (b) 19 differentially expressed genes between MITF^High^ and MITF^Low^ that best correlate with survival. (c) Heatmap showing correlation between the indicated genes and MITF or the Verfaillie et al invasive or proliferative gene expression signatures and Rambow et al signatures. (d) Heatmap showing relative expression of the 19 gene signature in the TCGA melanoma cohort ranked by risk. (e, f) Kaplan–Meier plots showing differential survival in the TCGA melanoma cohort between those with high versus low expression of the 19 gene signature comprising 7 MITF bound and correlated genes (e) and 12 MITF‐bound and anti‐correlated genes (f). (g) Expression of 8 of the 19 genes in the high versus low risk groups. (h) Kaplan–Meier plot showing differential survival in the TCGA melanoma cohort between those with high versus low expression of FAM105A. (i) Kaplan–Meier plot showing differential survival in the TCGA melanoma cohort between those with high versus low expression of a gene expression signature associated with T‐cells

Elevated expression of MITF is linked to decreased immune cell infiltration and moderately poorer survival. We found that 474 MITF‐bound genes whose expression correlates with MITF are also significantly correlated with survival. This includes 234 and 240 genes that are positively or negatively correlated with MITF expression respectively that as gene sets predicted survival (Figure 5a) better than MITF alone (Figure 1f). Using a Lasso Cox regression model, we were then able to generate a list of 19 MITF‐bound genes (Figure 5b) that as a gene set were best able to predict survival. The relationship between these genes and MITF or the Verfaillie et al. proliferative or invasive gene expression signatures, as well as two signatures previously generated in Rambow et al. (2015), are shown in Figure 5c. A risk score was calculated for each TCGA melanoma sample based on the 19 gene signature and samples dichotomized into high and low risk. The relative expression of each gene in the TCGA melanoma cohort ranked by risk is shown in Figure 5d. Kaplan–Meier curves showing survival probability based on the prognostic value of the expression of the genes set corresponding to the 7 genes bound and correlated with MITF are shown in Figure 5e and that for the gene set corresponding to the 12 genes bound and anticorrelated with MITF in Figure 5f. The expression in the high versus low risk groups of a subset of the 19 genes is shown in Figure 5g. Notably, the expression of just one of these genes, *FAM105A* (*OTULINL*), encoding an inactive endoplasmic reticulum‐associated ubiquitin thioesterase expressed predominantly in the low risk (MITF^Low^) group, represented a robust predictor of survival (Figure 5h). Indeed, as a predictive marker for survival expression of *FAM105A* (*p* = 4e‐7) outperformed T‐cell infiltration (*p* = 2.96e‐5), a frequently used marker for survival (Figure 5i).

### 3.4 MITF, the β‐catenin pathway, and immune cell infiltration

**FIGURE 6:**
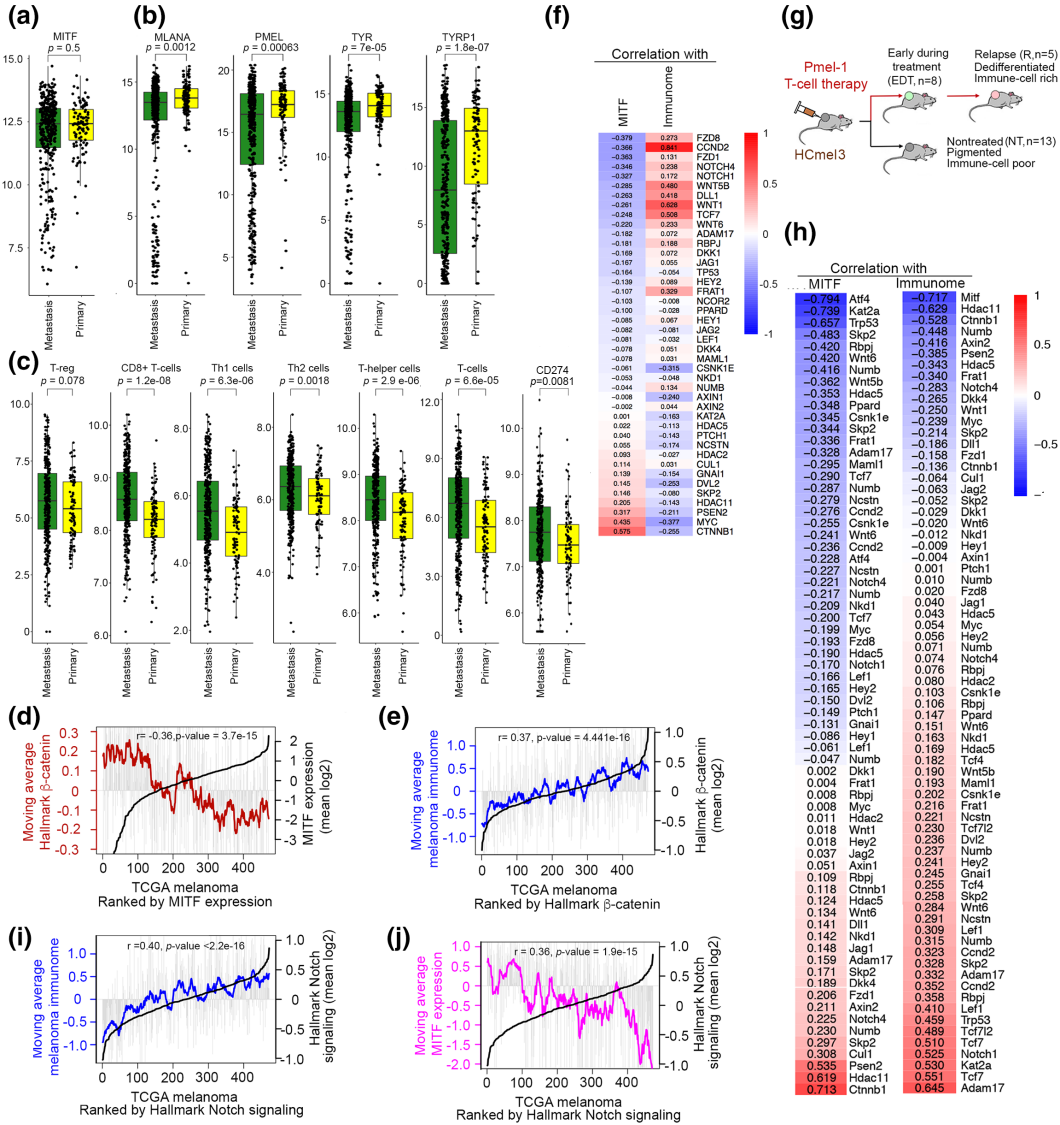
Expression of melanogenic genes and T‐cell infiltration in melanoma primaries and metastases. (a‐c) box and whisker plots showing expression of indicated genes (a, b) or signatures of specific immune cell types (c) in melanoma primaries versus metastasis in the TCGA melanoma cohort. (d) TCGA melanomas ranked by MITF expression. Grey bars indicate expression of the HALLMARK β‐catenin signaling genes set in each tumor, with the brown line the moving average of expression in every 20 tumors. (e) TCGA melanomas ranked by expression of the HALLMARK β‐catenin signaling gene set, with expression corresponding to the melanoma immunome in each tumor indicated by grey bars and moving average of the immunome in blue. (f) Heatmap showing correlation in the TCGA melanoma cohort between expression of individual genes in the HALLMARK β‐catenin gene set and MITF or the immunome gene expression signature. (g) Schematic showing use of adoptive T‐cell therapy in subcutaneous mouse melanomas. (h) Heatmaps showing correlation in murine melanomas between expression of individual genes in the HALLMARK β‐catenin gene set and MITF or the immunome gene expression signature. (i, j) TCGA melanomas ranked by the HALLMARK notch signaling gene set. The melanoma immunome (i) or MITF (j) expression is indicted by the grey bars and moving average of each by the blue and pink lines respectively

MITF^Low^ tumors correlate with increased survival. While there are several plausible explanations why this might be, one possibility is that the high immune cell infiltration in tumors with reduced MITF expression (Figure 1f‐h) favors survival. Whether MITF itself plays a role in regulating the immune response is not well understood (Ballotti et al., 2020). Recent evidence suggests that MITF can suppress NK‐cell‐mediated killing of melanoma cells (Sanchez‐Del‐Campo et al., 2021) and controls chemokine expression leading to reduced tumor immune infiltration in vivo (Wiedemann et al., 2019). Our preliminary analysis of TCGA melanomas confirmed that MITF^Low^ tumors exhibited increased immune cell infiltration (Figure 1h). However, we noted that while MITF expression was similar in primary tumors and metastases (Figure 6a), a number of well‐characterized MITF target genes (e.g., *TYR*, *TYRP1*, *MELANA*, *PMEL*) were significantly more highly expressed in primary melanomas than in metastases (Figure 6b). This observation suggested that while *MITF* mRNA levels were similar, MITF activity was higher in primary tumors. Consistent with this, immune infiltration was significantly higher in metastases than primary tumors (Figure 6c), although elevated CD274 (PD‐L1) expression in metastases was likely to suppress any antimelanoma response arising from increased immune cell infiltration. Thus, while *MITF* mRNA levels might contribute to controlling the immune response to melanoma, other factors, for example MITF post‐translational modifications or expression of MITF cofactors, must also be important.

Previous work from two independent groups has indicated that elevated β‐catenin signaling suppresses immune infiltration and consequently it has been suggested that this pathway contributes to a failure of antitumor immunity (Nsengimana et al., 2018; Spranger et al., 2015). Since *MITF* is a β‐catenin target gene (Dorsky et al., 2000; Takeda et al., 2000; Widlund et al., 2002), and β‐catenin acts as an MITF cofactor in regulating gene expression (Schepsky et al., 2006), this would make sense with both high β‐catenin signaling correlating with high MITF activity and low immune infiltration. However, we noted that if instead of using a subset of genes implicated in β‐catenin signaling, as in the previous studies (Nsengimana et al., 2018; Spranger et al., 2015), we used the Hallmark β‐catenin signaling gene set, then β‐catenin signaling anti‐correlated with MITF (Figure 6d) and significantly correlated with the degree of immune infiltration (Figure 6e). Significantly, the examination of individual genes in the Hallmark β‐catenin signaling gene set revealed a significant correlation between β‐catenin (CTNNB1) and MYC mRNA expression and MITF (Figure 6f). However, the expression of many genes within the Hallmark β‐catenin signaling gene set anticorrelated with MITF and correlated with the immunome gene expression signature.

The TCGA melanoma samples contain a wide range of tumors with varying degrees of immune cell infiltration ranging from high “hot” to low “cold” tumors. By contrast, mouse melanomas are relatively homogeneous. Nevertheless, we asked whether the results would be recapitulated in a mouse model using independent gene‐array‐based datasets (Landsberg et al., 2012; Reinhardt et al., 2017) derived from a mouse model of adoptive T‐cell therapy. This HgfxCdk4 mouse model (Figure 6g), and subcutaneous injection of derived murine HCmel13 melanoma cells gives rise to tumors MITF^High^ differentiated tumors that are poorly infiltrated with immune cells. However, adoptive T‐cell therapy based on targeting cells expressing the differentiation antigen Pmel‐1 leads to initial tumor regression but subsequent relapse with immune cell‐rich tumors exhibiting loss of Pmel‐1 antigen and consequently resistance to the T‐cell‐based therapy (Landsberg et al., 2012). To recapitulate the heterogeneity of immune cell infiltration seen in human melanoma cohorts, including the human TCGA melanomas, we pooled gene expression data derived from tumors from untreated versus treated mice and again asked whether the genes within the Hallmark β‐catenin signaling gene set correlated either with MITF or the immunome gene expression signature in the different tumors. Note that as the mouse model uses gene arrays, the expression of some genes is interrogated by more than one probe that in some cases may give different results eg that for the *Numb* gene. However, as we do not know the origin of the variable results which might arise owing to differential splice isoforms (there are 4 variants for *Numb* for example) or from other unknown causes, we present the results for all probes rather than cherry‐picking some that might fit a specific narrative. The results (Figure 6h) indicate that, as in the human melanomas, Mitf correlated well with expression of *Ctnnb1*, *HDAC11* and *Psen2*, but unlike the human tumors *Myc* was not correlated with *Mitf*. Similarly most of the genes whose expression anticorrelated with *MITF* in the human tumors also anticorrelated with *Mitf* in the mouse model, whereas the immunome in human tumors anticorrelated with *CTNNB1*, *PSEN2* and *HDAC11* expression, the same genes anticorrelated in the mouse model. Nevertheless, as in the human tumors, many of the genes in the Hallmark β‐catenin signaling gene set correlated with the immunome signature in the mouse tumors.

However, we also noted that the Hallmark β‐catenin signaling gene set included many genes implicated in Notch signaling, including *NOTCH1* and *NOTCH4*, their ligand *DLL1*, *RPBJ* that targets Notch intracellular domain to DNA, and the Notch pathway effectors *HEY1* and *HEY2*. Since Notch signaling has a key role in immune regulation in the tumor microenvironment (Colombo et al., 2018), we also used the Hallmark Notch signaling gene set to rank the TCGA melanoma cohort and found a significant correlation between Notch signaling and immune infiltration in the TCGA melanomas (Figure 6i). Similarly, Notch signaling inversely correlated with MITF (Figure 6j). Similar results were also obtained in the mouse tumors (Figure 6h) where many Notch pathway genes (*Notch1*, *Rbpj*, *Hey2*, *Maml*) were positively correlated with the immunome.

## 4 DISCUSSION

Over the recent years, substantial evidence has accumulated to indicate that MITF plays a broad role in coordinating melanocyte and melanoma biology beyond its function in promoting melanocyte cell identity and regulation of melanosomal genes (Goding & Arnheiter, 2019). In particular, it can drive pro‐proliferative gene expression and proliferation‐associated metabolism while at the same time it suppresses invasion. Moreover, in vivo, expression of MITF in melanoma anticorrelates with immune infiltration (Riesenberg et al., 2015; Tirosh et al., 2016). How MITF coordinates so many aspects of cell biology remains poorly understood.

Most studies have used cell lines in vitro to identify the repertoire of genes regulated by MITF, for example by using ChIP‐seq combined with siRNA‐mediated MITF depletion to identify MITF bound and regulated genes. However, these studies are limited given the microenvironment of cells in monolayer culture is very different from that encountered in vivo and depletion of any mRNA may be associated with off‐target effects and may vary significantly in different cell lines. By contrast, in melanomas, the microenvironment within tumors which is known to affect MITF activity, is complex and varies within and between tumors. Moreover, although in vivo MITF expression may be correlated with expression of a putative target gene, depletion of MITF to confirm regulation is difficult. Here instead, we chose to try to identify genes that are likely regulated by MITF based on correlations in both cell lines and in vivo, combined with ChIP‐seq data from melanoma. The results confirmed in both cell lines and in tumors that MITF expression correlates with a gene expression signature associated with proliferation and anticorrelates with an invasive signature. We also noted, that in addition to the anticipated correlation between MITF and pigmentation genes, the cell cycle and metabolism, we found MITF linked to protein translation and ribosome biogenesis. Since elevated protein translation is necessary to support proliferation and suppress senescence, this observation may partly explain the cell cycle defects (Carreira et al., 2006) and senescence (Giuliano et al., 2010) observed on MITF depletion. By contrast, the genes that are bound by MITF and that correlate in vivo and in vitro with low MITF expression are primarily associated with production of extracellular matrix (ECM) as noted previously (Dilshat et al., 2021). This suggests that one role for MITF may be to suppress the generation of ECM in proliferative cells. This is important as ECM signaling via integrins and SRC can modulate the activity of the hippo signaling pathway effector TEAD (Ma et al., 2019) that plays a key role in the invasive gene expression network and resistance to MAPK pathway inhibitors (Verfaillie et al., 2015).

The identification of ECM genes bound and anticorrelated with MITF expression extends substantially the potential repertoire of MITF repressed genes, and matches the recent observation that MITF knockout cells upregulate expression of a number of ECM‐related genes (Dilshat et al., 2021). How MITF might activate some genes or repress others has been a key unresolved question. While it is possible that MITF differentially associates with cofactors linked to repression or activation at different genes, our results suggest a different mechanism may operate. First, we noted that for potentially repressed genes a smaller proportion of MITF‐binding sites were associated with promoters. This observation indicates that the position of binding sites may be an important determinant for whether MITF activates or represses gene expression. For example, binding in an intron or intergenic region could act to decoy the transcription machinery away from the promoter of a nearby gene leading to reduced transcription initiation. Second, while genes bound and positively correlated with MITF contained the classic CACGTG MITF recognition motif, genes whose expression anticorrelated with MITF tended to contain JUN/FOS (AP1) or ATF3 sites, as well as MITF sites. AP1 activity has been strongly linked to the undifferentiated, invasive and drug‐resistant MITF^Low^ phenotype (Riesenberg et al., 2015; Verfaillie et al., 2015). Since direct binding by bHLH‐LZ factors such as MITF is restricted to E‐box motifs, it seems possible that any repression of genes containing AP1 or ATF3 sites is indirect and may reflect MITF interaction with these transcription factors via protein–protein interactions to prevent transcription activation. This interpretation is consistent with the interactions detected between MITF and both JUN and ATF3 in our mass spectrometry data. Only when MITF expression is decreased, for example in response to hypoxia, nutrient limitation or inflammation, would the activity of AP1and ATF3 be derepressed. However, our conclusions are limited by the absence of data on the location of AP1 binding sites in the same cells as those used for the MITF‐ChIP‐seq. Note, we also detected an MITF‐TFAP2 interaction that presumably would promote transcription of coregulated differentiation associated genes. Why MITF interaction with some transcription factors might lead to repression, and yet interaction with others is associated with gene activation requires further exploration, but it is possible that the position of the MITF binding sites relative to the transcription startsite, enhancers, or positioned nucleosomes may play a key role.

The potential of MITF to repress AP1‐regulated genes (Riesenberg et al., 2015) is important. Acute MITF loss can sensitize cells to tumor necrosis factor α (TNFα) (Riesenberg et al., 2015), a key inflammatory signal that is implicated in melanoma de differentiation and resistance to adoptive T‐cell therapy (Landsberg et al., 2012). Significantly, the induction of many cytokine and chemokine genes in melanomas is dependent on AP1, consistent with increased immune cell infiltration in MITF^Low^ tumors (Riesenberg et al., 2015; Tirosh et al., 2016). Here, we extend these observations to dissect the repertoire of infiltrating immune cells in MITF^High^ versus MITF^Low^ tumors and find that immune infiltration correlates with low MITF activity, and that MITF target gene expression, but not MITF mRNA, is reduced in metastases versus primary tumors. However, although metastases have a higher immune cell infiltration, they also express more CD274/PD‐L1 suggesting that metastases may exhibit a more immune suppressed environment. In addition to low MITF, we also find that immune cell infiltration correlates with high Notch pathway signaling. By contrast, while we can reproduce the inverse correlation between a restricted subset of β‐catenin target genes and immune infiltration observed previously (Nsengimana et al., 2018; Spranger et al., 2015), our observations suggest that any involvement of β‐catenin may be more nuanced, with the Hallmark β‐catenin signaling gene set correlating with immune infiltration. One interpretation of this observation is that TCF4/TCF7L2 may target β‐catenin to a distinct set of target genes in MITF^Low^ cells, but that in the MITF^High^ phenotype β‐catenin may use other targeting factors such as LEF1 (Eichhoff et al., 2011) or MITF itself (Schepsky et al., 2006). While this may be speculation at present, it nevertheless represents a testable hypothesis that is currently being explored.

## AUTHOR CONTRIBUTIONS

JC performed the analyses with the exception of those for the mouse tumors that were performed by MH. CRG and FMB provided supervision. J‐PL conducted and analyzed all mass spectrometry experiments. CRG and JC prepared the manuscript. All authors read and approved the final manuscript.

## CONFLICT OF INTEREST

The authors declare they have no competing interests.

## Supporting information


Figure S1.

Figure S2.

Figure S3.

Figure S4.

Table S1.

Table S2.

Table S3.

## Data Availability

The analyses presented used the following melanoma datasets: CCLE (https://sites.broadinstitute.org/ccle/); the clinical expression data from TCGA melanoma cohort. TCGA melanoma RNAseq data was accessed through the cBioportal for Cancer Genomics (http://www.cbioportal.org) using the R‐based package CGDS‐R. We retrieved mRNA expression (RNA Seq V2 RSEM) values for 473 melanoma samples. The datasets for the mouse HCmle3 tumors are accessible through GEO under the accession numbers GSE40213 and GSE99925. All MS files used in this study were deposited to MassIVE (http://massive.ucsd.edu) and ProteomeXchange (http://www.proteomexchange.org/), assigned the accession number MSV000089109, and PXD032772 can be accessed at ftp://msv000089109@massive.ucsd.edu.
