# Supplementary figures and images for: The MITF regulatory network in melanoma
Source: Pigment Cell Melanoma Res. 2022 Jul 9;35(5):517–33. doi: 10.1111/pcmr.13053 (PMC9545041; doi:10.1111/pcmr.13053)

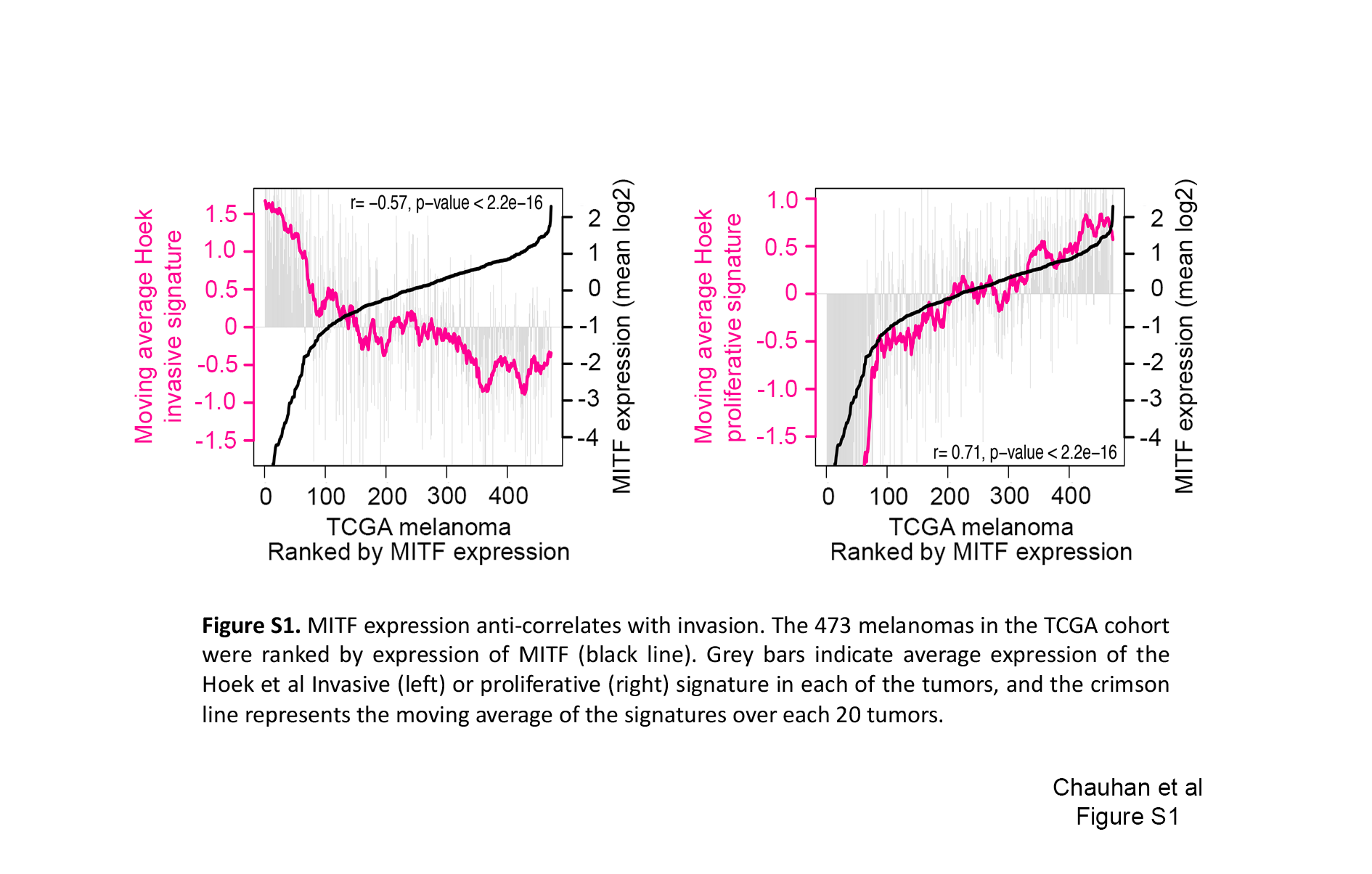

Supplement: Supplementary file 1 — Figure S1. [file PCMR-35-517-s006.tif]

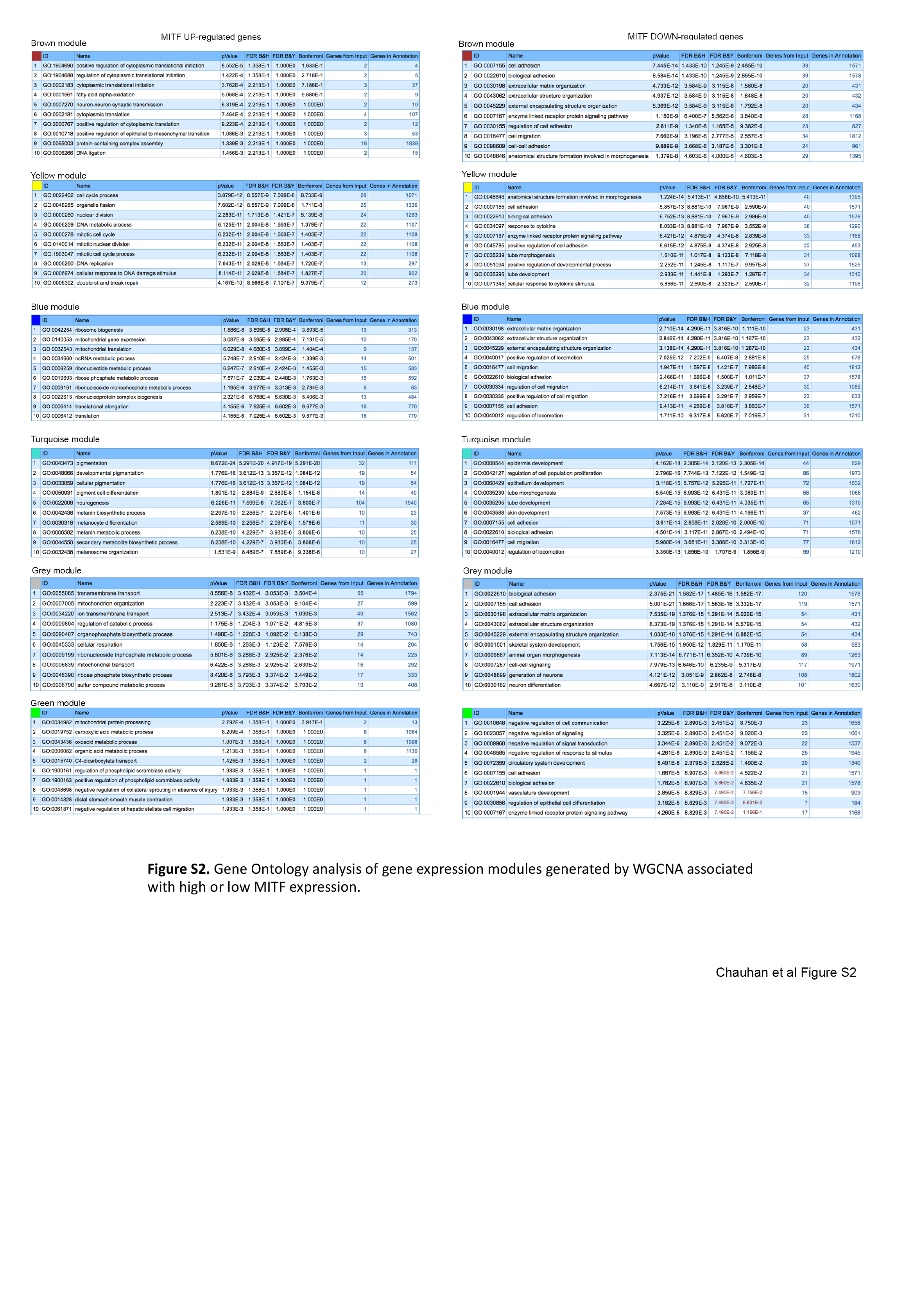

Supplement: Supplementary file 2 — Figure S2. [file PCMR-35-517-s004.tif]

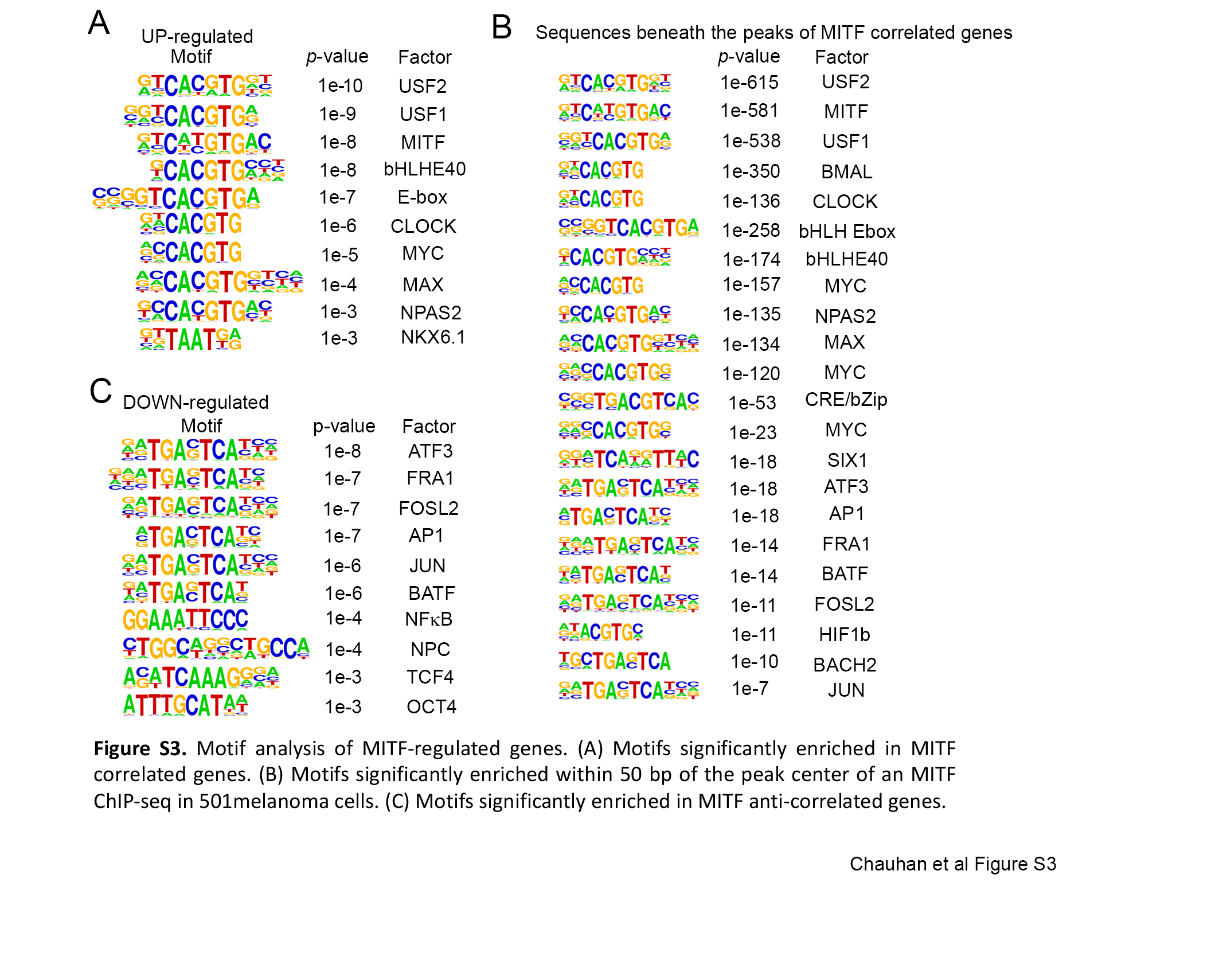

Supplement: Supplementary file 3 — Figure S3. [file PCMR-35-517-s002.tif]

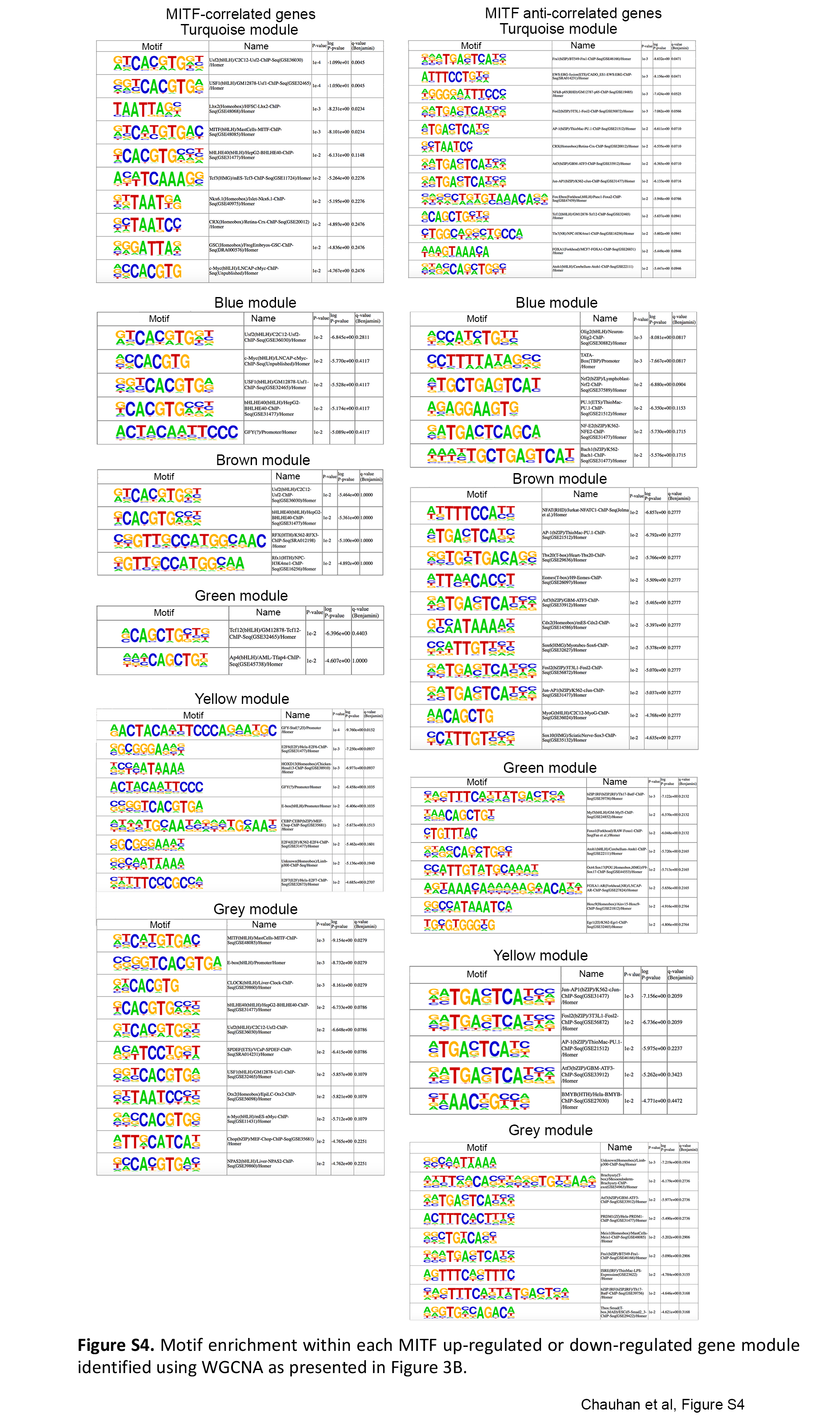

Supplement: Supplementary file 4 — Figure S4. [file PCMR-35-517-s003.tif]
